# Recent Advances in the Polish Research on Polysaccharide-Based Nanoparticles in the Context of Various Administration Routes

**DOI:** 10.3390/biomedicines11051307

**Published:** 2023-04-28

**Authors:** Mateusz Młynek, Jakub Waldemar Trzciński, Tomasz Ciach

**Affiliations:** 1 Faculty of Chemical and Process Engineering, Warsaw University of Technology, Waryńskiego 1, 00-645 Warsaw, Poland; 2 Centre for Advanced Materials and Technologies CEZAMAT, Warsaw University of Technology, Poleczki 19, 02-822 Warsaw, Poland

**Keywords:** drug administration, drug delivery system, polysaccharide nanoparticle, alginate, chitosan, dextran

## Abstract

Polysaccharides are the most abundant polymers in nature. They exhibit robust biocompatibility, reliable non-toxicity, and biodegradable character; thus, they are employed in multiple biomedical applications. The presence of chemically accessible functional groups on the backbone of biopolymers (amine, carboxyl, hydroxyl, etc.) makes them suitable materials for chemical modification or drug immobilisation. Among different drug delivery systems (DDSs), nanoparticles have been of great interest in scientific research in the last decades. In the following review, we want to address the issue of rational design of nanoparticle (NP)-based drug delivery systems in reference to the specificity of the medication administration route and resulting requirements. In the following sections, readers can find a comprehensive analysis of the articles published by authors with Polish affiliations in the last few years (2016–2023). The article emphasises NP administration routes and synthetic approaches, followed by in vitro and in vivo attempts toward pharmacokinetic (PK) studies. The ‘Future Prospects’ section was constructed to address the critical observations and gaps found in the screened studies, as well as to indicate good practices for polysaccharide-based nanoparticle preclinical evaluation.

## 1. Introduction

Dozens of new therapeutics are approved by the United States Food and Drug Administration (FDA) each year. Even though the number of new drugs discovered is recently constant, each new pharmaceutically active substance must be evaluated on absorption, distribution, metabolism, excretion, and toxicity (ADMET) criteria, which are not always straightforward. According to Ranade V.V. and Hollinger M.A., editors of the second edition of *Drug Delivery Systems*’ [1], there are three basic approaches to address the drug delivery absorption problem (formulation, pro-drug, and analogue approach). The first is the easiest and cheapest of the above categories due to limited synthetic efforts. Among different types of drug delivery systems and dosage forms, e.g., microemulsions, hydrogels, microcapsules, and hollow fibres, one can distinguish nanoparticles. They can be further divided into a few groups: inorganic NPs, dendrimers, liposomes, polymeric NPs, and micelles, which include polysaccharide-based NPs [2].

Modern medicine entrenches from a span of athenaeum of naturally occurring products, where nearly 25% of biologically active compounds are derived from natural sources [3]. Instead, the diversity of carbohydrate natural building blocks, especially bearing polymer relevance, is limited chiefly to several saccharides: glucose, saccharose, galactose, and fructose [4]. These mono-sugar subunits assemble via α- or β-glycosidic bonds, which affect their cognisance like homopolysaccharides or homoglycans, which instead strongly entails their stability and applicability in living organisms. Thus, polysaccharides’ formation can be classified according to several aspects: the occurrence, structure, or application. Nevertheless, their chemical structure is predominant in nano-based drug delivery systems’ classification. Therefore, the mono-sugars’ sub-chain functionalisation takes importance, like the amine side group in chitin and hyaluronic acid, sulfonyl moiety in heparin or carrageenan, or the carboxyl group in alginate and pectin [4]. The presence of several functional groups in the polysaccharide backbone is often the natural strategy to overcome adverse environmental conditions, such as chondroitin being exposed to high shear forces in the bone joints [5]. By dint of their complex structure, the polysaccharides can be priceless materials due to their immunoregulatory properties, cell receptor binding ability, and biological information transporters [6].

As has been proven by over 20 years of research, nanoparticles improved conventional medicine with high specificity via active targeting, intensified loading capacity, suitable drug-release profiles, longer half-life circulation time of a drug, and higher bioavailability in reference to a free drug molecule. Natural polymers gained special attention among the used materials due to their high biocompatibility and biodegradability and the possibility of decorating a polymeric backbone with several molecules (drugs, luminophores, targeting agents, and hydrophobic domains) to increase the efficiency of payload delivery. Polysaccharides are extensively studied as potential polymeric materials for drug delivery systems, since their chemical compatibility enhances the therapeutic substances’ introduction to the human body [7,8]. Their ability to form crystalline or amorphous phases is one of the most distinctive properties of biomedical applications, strongly affecting the therapeutic efficacy [9]. Instead, this allows using polysaccharide nanoparticles for sustained release via several mechanisms, such as enzymatic degradation, swelling, or bursting. All their biological activities (e.g., antitumor, antiobesity, or antidiabetic) are strictly related to their side chain modifications.

**Figure 1:**
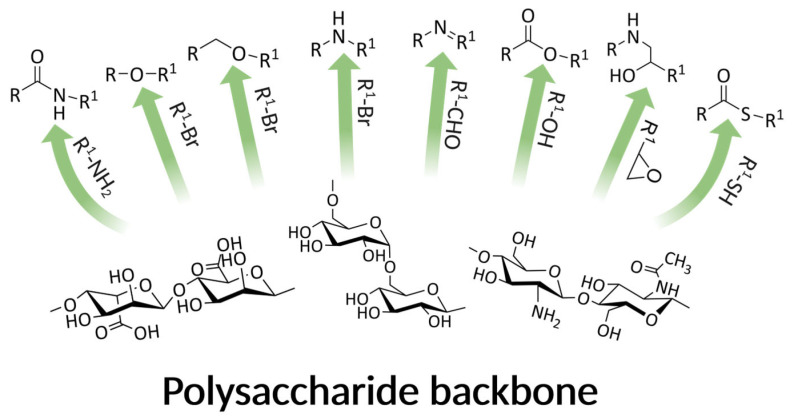
Polysaccharide possible chemical modifications (R = saccharide group). Arrows indicate a possible modification route of each polysaccharide substrate-specific functional group (-OH, -NH_2_, or -COOH) to the corresponding product by the reaction with reagents drawn next to the arrows (namely, amine, alkyl halide, aldehyde, alcohol, epoxide, or thiol).

Although nanoparticles’ properties can be easily modified (e.g., size range, surface charge, material biocompatibility, attachment of targeting molecules), their design must meet many demanding criteria, which is essential for efficient and safe drug delivery to a specific destination. Both top-down and bottom-up techniques can be successfully used for nanoparticles’ preparation [7,10]. A wide range of possible polysaccharide modifications made them ideal basal materials for NP synthesis. The possible synthetic approaches toward common polysaccharides’ chemical moieties transformation are presented in Figure 1.

The following section of this review is constructed to give the reader a brief description of drug administration routes and their implications on nanoparticles’ properties. Finally, the third part of the article discusses the technical aspects of polysaccharide-based nanoparticle synthesis based on Polish research articles published in recent years (2016–2023). While reviewing the selected papers, the main emphasis was placed on two aspects: the authors’ approach to biomaterial synthesis and the scope of the conducted biological studies.

## 2. Anatomical Drug Administration Routes and Their Implications for the Design of Nanoparticles

**Figure 2:**
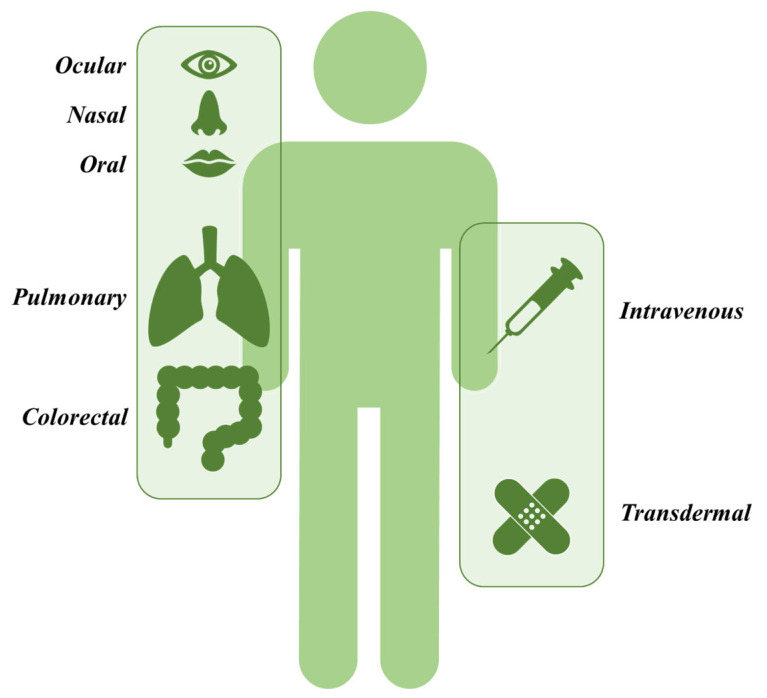
Symbolically presented anatomical routes of drug administration in the human body that could be considered for nanoparticle-mediated active substance delivery.

The human body has many anatomical drug administration routes (Figure 2). Among others, one can distinguish the gastrointestinal tract (GIT) and transdermal (TD), nasal (NL), ocular (OC), pulmonary (PM), or intravenous (IV) routes of administration. Each of them presents both advantages and disadvantages that must be considered prior to nanometric DDS design. The following sections will discuss the most important aspects of each delivery path regarding nanocarrier properties that need to be considered.

Herein, we discuss the characteristics of polysaccharide-based NPs that should be considered to overcome difficulties associated with each route of administration. Table 1 compares both benefits and problems identified for oral, nasal, ocular, transdermal, pulmonary, rectal, and intravenous drug applications.

From the perspective of a polysaccharide-based nanoformulation designed for oral administration, it is crucial to remember the harsh conditions prevailing in the GIT. Although the majority of medicines are delivered orally, they often lose a high percentage of their activity due to partial hydrolysis or clearance [11]. Another crucial obstacle to oral administration is the first-pass effect associated with the hepatic portal system. A profound amount of medicine absorbed in the intestine is metabolised and secreted; hence, the bioavailability of orally applied drugs is significantly decreased. Preselected basal material for a drug nanocarrier should be resistant to hydrolytic enzymes and high stomach acidity. The utilisation of polysaccharides as a matrix for nanoparticle synthesis exhibits considerable resistance to the abovementioned due to their unique structural properties. Naturally occurring polysaccharides are not easily accessible for mammalian glucosidases, e.g., dextran consisting of glucose subunits connected mainly by α-(1,6)- and α-(1,3)-glycosidic bonds or chitosan containing β-(1,4)-linkages between D-glucosamine and N-acetyl-D-glucosamine units [12,13]. While nanoparticles design focuses mainly on the delivery method, it is crucial to remember the ADMET properties. For orally administered formulations, the first of the ADMET properties, absorption of the nanoparticulate system, is conventionally tested via in vitro assay in Caco-2 cells [14,15].

**Table 1:** Advantages and disadvantages of main medicine administration routes in humans.

Administration Route	Benefits	Difficulties	Ref.
Oral	possibility for self-administration dose up- or downscaling ease of adjusting the frequency of administration plenty of available medicine formulations patient preferences non-invasiveness convenience of discontinuation of drug intake	the hydrolytic activity of digestive enzymes and the GIT microbiome pH variations uncontrolled systemic drug concentration possible irritation of intestinal epithelium transit time differs from patient to patient and is dependent on food intake buccal mucosa irritation	[12,16,17,18,19,20]
Nasal	fast relief in allergic conditions avoidance of high first-pass metabolism low level of enzymes and short exposure time increased permeability of nasal mucosa resulting in rapid absorption possibility of BBB circumvention ease of self-administration	nasal mucosa oedema bioavailability highly dependent on the nasal secretion influence of the environmental conditions (temperature and humidity)	[12,21]
Ocular	conventional route for ophthalmic medical prevention lack of first-pass effect metabolism topical application provides ease of self-administration systemic side effects are minimised lower drug doses due to localised delivery	high tear-fluid turnover rate drug leakage due to nasolacrimal drainage invasive intravitreal injections can lead to retinal detachment or haemorrhage bioavailability affected by eye static and dynamic barriers blinking complicates self-administration and drug distribution corneal cells are barely permeable to drugs elevated ocular irritation risk presence of ocular lysosomal enzymes	[22,23,24]
Transdermal	convenience of discontinuation of drug intake possibility and ease of self-administration avoidance of high first-pass metabolism long drug release time well-examined permeation enhancers desired site of application and lower systemic exposure yielding reduction in side effects therapeutic peptides and proteins sensitive to digestive enzymes can be delivered direct targeting of skin disorders	challenging administration of hydrophilic drugs difficulties with drug absorption in in vitro studies slightly acidic physiological pH (4.5–6.0) unique dermal microbiome and pH conditions strictly connected with individual demographics and lifestyle limitations in 3rd generation enhancers use due to local skin sensitivity (e.g., around the eyes) possible allergic skin reactions effective permeant should be lower than 500 Da	[12,25,26]
Pulmonary	convenience of discontinuation of drug intake avoidance of high first-pass metabolism a thin cellular layer to be crossed to reach the systemic blood circulation large surface area improves the drug absorption ease of self-administration/inhalation direct lung disease treatment (e.g., COPD, asthma, lung cancer, ALI, and IPF) possible systemic administration due to high vascularisation of alveoli	hardly permeable ciliated surfactant-covered alveolar surface absorption needs to be quick enough to avoid exhalation of drug presence of mucus gel impedes drug deposition and absorption due to clearance mechanisms, deposited particles are constantly swept up peptide and protein enzymatic degradation by peptidase require a separate device for application possibility of BBB bypass	[12,27,28,29]
Colorectal	convenience of discontinuation of drug intake first-pass metabolism can be omitted applicable for patients with swallowing problems (e.g., paediatric and geriatric) lower enzymatic activity in the rectum in comparison to other parts of GIT ease of rectal self-administration (suppository, rectal foam, or gel)	irritation with a risk of proctitis, ulceration, or bleeding drug retention is dependent on gastric emptying rate (especially low retention during diarrhoea) non-specific drug interaction with faecal matter difficult permeability for hydrophilic drugs	[12,30]
Intravenous	lack of necessity to pass physical or biological barriers avoidance of high first-pass metabolism controlled systemic drug concentration rapid onset of action (appropriate for emergency incidents) immediate drug concentration peaks (1–2 h) an efficient way of vaccine administration (peptides, proteins, and nucleic acids)	invasive administration method possibility of non-selective systemic effects pain at the injection site problem with sterility (risk of infection) high dilution rate fast clearance by kidneys require the assistance of qualified personnel haemolysis risk	[12,31]

Abbreviations used: ALI, acute lung injury; BBB, blood–brain barrier; COPD, chronic obstructive pulmonary disease; GIT, gastrointestinal tract; IPF, idiopathic pulmonary fibrosis.

Intranasal drug administration attractiveness is mainly attributed to fast topical action and the possibility of passing the blood–brain barrier via olfactory and trigeminal nerves [32]. Applying polymeric- or polysaccharide-based NPs increases mucoadhesion in the nasal cavity and retention time, consequently improving drug bioavailability [21]. Moreover, it was reported that drugs delivered intranasally are absorbed mainly via the aqueous channel of the mucous membrane [12]. Therefore, the NPs designed for this delivery route should exhibit hydrophilic properties and excellent stability in an aqueous solution. The abovementioned pumps are not the only possibilities for nanometric DDS transportation, as they can be internalised via endocytosis and pinocytosis or diffusion depending on the size and molecular weight [33]. Since there is no accurate in vitro model for nasal DDS absorption testing, ex vivo models are being applied [34,35].

**Figure 3:**
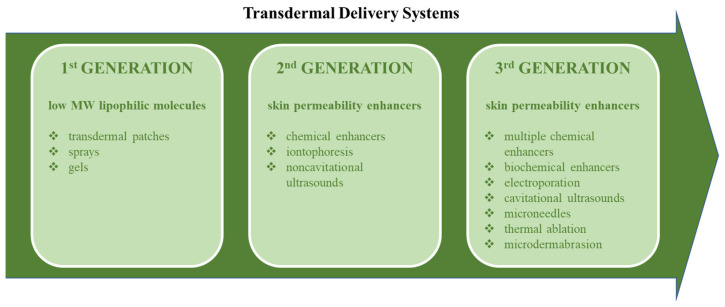
The classification of transdermal delivery systems into three main generations (based on the *Nature Biotechnology* article [25]). Each presented column represents a separate transdermal DDS generation with the scope of applications and the corresponding examples.

Thanks to rapidly increasing DDS development and market transformation, nearly 9% of drugs approved by the United States Food and Drug Administration (FDA) by 2018 were dedicated to cutaneous administration [36]. It was proven that the drug concentration profile obtained by a transdermal application could be more appropriate than intravenous, thanks to the long-lasting plateau phase [37]. There are several factors determining effective transdermal drug absorption. The first one, the diffusion through the skin layers, can be described by Fick’s first law. To determine the efficiency of skin permeation, the following parameters should be measured: partition coefficient, diffusion coefficient, and diffusion path length [38,39]. On the other hand, once successfully absorbed into the skin, the drug or nanoformulation is exposed to enzymatic activity. Numerous enzymes were identified to catalyse cutaneous xenobiotic reactions (i.e., cyclooxygenases, esterases and amidases, epoxide hydrolases, proteases, glutathione S-transferase, and N-acetyltransferases) [40]. It should be emphasised that they can have either harmful (degradation) or positive (drug release) effects depending on the drug structure and formulation design. Transdermal DDSs are classified into three main generations, presented in Figure 3. Each dosage form pointed out in the graph has room for nanoparticle application.

Thanks to the presence of different functional groups on their surface, polysaccharide-based nano vehicles can be easily modified by adding permeability enhancers. This approach, together with active transport methods through the stratum corneum (e.g., electroporation, cavitation ultrasounds, microneedles, and thermophoresis), can extensively increase delivery efficiency [41]. In the context of NP transdermal permeability, it is essential to determine partition or distribution coefficients (logP or logD, respectively) that should fit in the range from 1 up to 3. This enables effective diffusion of the formulation through the skin without the risk of retention in the lipid portion of the skin layers [42]. Applying polysaccharide-based nanoparticles acting like surfactants may enhance cargo stability, solubility, and permeability [43]. In addition, polysaccharide resistance to different microbial hydrolytic enzyme types makes them a tremendous basal material for topical DDSs. Among others, chitosan is the most prominent of the glycosidic biopolymers due to the positive zeta potential, which is the opposite of that exhibited by the dermal surface. This phenomenon implies strong adhesion of chitosan-based nanomaterials with skin [44]. Both the efficacy and safety of a nanoformulation can be determined using in vitro and in vivo tests. There are several possibilities for testing TD formulations in vitro, for example, cell cultures of keratinocytes and 3D cell culture. Each method has its own advantages and disadvantages, and they have been briefly summarised by numerous review articles [40,45,46].

The pulmonary route of administration is characterised mainly by a sizeable alveolar area available and a high amount of capillary beds together, offering conditions for effective absorption. Depending on the size of nanocarriers, they can be transported through the alveoli barrier by one of three possible routes: (1) transcytosis (>40 kDa), (2) transporting proteins, or (3) paracellular absorption (<40 kDa) [47]. As the alveolar surface is covered with thick polymeric mucus and a lung surfactant produced by type II cells, it seems vital to test whether and how the designed NPs’ absorption is affected by the abovementioned factors [48,49]. Difficulties also arise when it comes to the studies with cell lines, as there are several types of epithelial cells in lungs (e.g., goblet cells, basal cells, ciliated cells, and brush cells), and the thickness of the layer differs significantly [50]. There is ongoing research on new advanced cellular models for in vitro studies, but due to the complexity of the airway tracts built, more time is needed to see the effects [51,52]. It must be mentioned that there are strict aerodynamic requirements for effective lung inhalation. Three main mechanisms are responsible for the efficacy of nanometric DDS transportation into the lung: initial impact with the lung’s inner surface, gravitational sedimentation, and diffusion [29,53]. The last two mechanisms are highly correlated with particle size and shape. The most significant effect for NP deposition and absorption is prescribed to Brownian diffusion (for particles less than 0.5 μm). Once a free drug molecule, or one encapsulated in a nanocarrier, is successfully deposited on the gel layer, the absorption and clearance mechanisms occur. Most solid particles are trapped in the gel and move toward the pharynx due to cilia movements [54]. On the tissue level, it was proven that lipophilic molecules undergo passive transport, while hydrophilic particles cross via active transport or tight junctions. Unabsorbed drug particles can be either phagocytosed, enzymatically degraded, or circulate in the pulmonary tract [54]. In 2018 the FDA approved Arikayce^®^, a liposomal suspension, with amikacin (antibacterial aminoglycoside) as the active substance for the treatment of lung bacterial (Mycobacterium avium) disease [55]. Its success can be attributed to the long serum half-life locally in the lungs and strongly reduced systemic exposure. Moreover, it proves that a nanoformulation can effectively penetrate alveolar macrophages without affecting their function, facilitating further research on NPs for respiratory drug delivery. Polysaccharides revealing mucoadhesive properties are great candidates for DDS material, extending retention time. There are difficulties in choosing one applicable in vitro method for lung delivery route evaluation [48]. Unfortunately, animal models remain the most prominent way to test pulmonary drug administration, although the FDA seems to deviate from it [27,56].

Not so popular but bearing some advantages, the ocular route of administration also leaves space for nanotechnological improvements. As it is one of the most critical sensory organs, the human eye is protected by numerous physical and chemical barriers. The absorption of any medication applied via the ophthalmic route will be hampered by factors such as tear turnover, drainage, enzymatic activity, and environmental complexity [23]. The first physical barrier on the path of the topically administered drug is a tear film composed of a lipid layer followed by aqueous and mucin layers. Further, it meets the corneal epithelium with efflux pumps in the cell membrane. This thin cell layer allows for the absorption of hydrophobic particles but is hardly permeable to hydrophilic ones due to tight junctions [57]. Once the drug crosses the cornea, the drug encounters high osmotic pressure at the entrance to the vitreous body. When approaching the eyeball from the posterior side, medicine must penetrate the Bruch’s membrane, consisting of the choriocapillaris and retinal pigment epithelium [58]. The ocular pharmacokinetics are complex due to the sophisticated eye structure, but several simulation models for each compartment exist [59]. The PK parameters depend not only on the ocular administration site but also on the dosage form. For example, Urtii et al. used a simulation model based on the Quantitative Structure Property Relationship (QSPR) to predict the clearance of intravitreal drugs [60]. When it comes to animal models, the most common one is the rabbit model, allowing to obtain predictable parameters in humans [59,61]. Moreover, it is well established that xenobiotic transporters (e.g., efflux pumps, such as P-glycoprotein (P-gp), multidrug resistance protein (MRP), and breast cancer resistance protein (BCRP), and influx pumps, such as oligopeptide transporter (OPT), sodium-dependent multivitamin transporter (SMVT), and amino acid transporter (B(0,+)) play a significant role in ocular PKs. In summary, the drug or nanometric DDS must meet many criteria to circumvent the abovementioned barriers. Although many implants (e.g., drug-eluting contact lenses, microneedles, Durasert, Vitrasert, Retisert, NOVADURT, Renexus, etc.) to the anterior and posterior of the eyeball exist, the application is invasive, leading to eye irritation [22]. Nanotechnology brings some superiorities to ocular DDSs by improving drug solubility, controlling secretion, and increasing precorneal residence time, thus enhancing total bioavailability. Polymeric nanomicelles, characterised by a hydrophilic corona and lipophilic core, can be applied to ocular DDSs. Due to their amphiphilic character, they allow for better biological and physical layer penetration. A great example of a nanomicellar formulation is Cequa^®^ (Sun Pharmaceutical Inc.), which contains cyclosporine-A as an active substance for dry eye disease treatment [62]. The mucus covering the cornea and conjunctival tissues bears a negative charge due to the presence of mucins (high-molecular-weight glycoproteins). Thus, cationic polysaccharides and their derivatives (e.g., chitosan) are of great interest as materials for ocular drug-delivering NP synthesis [63]. Although in vitro ophthalmic cell cultures for toxicity testing are constantly transforming [64,65], animal models still function as the most accurate method for testing DDSs [66].

Despite it seeming undervalued, the colorectal tract for drug administration indicates a huge predominance in the systemic absorption rate. The colon is part of the intestine, where food and other ingested ingredients reside approximately 50 times longer than in the small intestine. For this reason, and the elevated blood flow rate through the absorptive epithelium, an administered drug can be effectively transported to the systemic circulation (from the lower rectum part) or the portal system (from the upper rectum part) [12]. The main limitation regarding medicine absorption process potency is a low volume of rectal fluid (1–3 mL). The applied formulation must dissolve quickly and efficiently to ease drug diffusion through the mucus and intake by epithelial cells [67]. Polysaccharide-based NPs functionalised with lipophilic moieties are promising candidates to overcome hydrophilic drug absorption problems in this part of the colorectum. Nanomicellar solutions would be of great interest due to their amphiphilic nature and already solubilised drug form. Unfortunately, even though successful drug or nanocarrier absorption is crucial, it is not sufficient to provide a therapeutic effect, as the drug can be excreted by cellular ABC transporters [61]. For comprehensive studies on ADMET properties in colorectal drug administration, animal models (mostly mice) can be applied [68,69]. Among others, the partition coefficient is a crucial parameter that characterises administered drug properties and can be useful in formulation applicability determination [70]. Even though the final part of the GIT exhibits little microbial enzymatic activity, it is worth evaluating its nanocarrier resistance. It is also significant to avoid formulations containing alcohols, as they can be oxidised by rectal microbiota to cancerogenic aldehydes, leading to toxic effects [71,72]. Available formulations (foams, suppositories, and enemas) can hardly penetrate deeper parts of the large intestine, making rectal administration more suitable for local action in the distal colon [30]. Rectal pH is within the range of 7–8 and is barely buffered [17]. For this reason, it should be considered whether an applied nanoformulation could alter the physiological pH value, leading to changes in drug absorption and enhancing the risk of rectal mucosa damage [70]. The neutral colorectal pH does not affect the pH-triggered drug carriers, resulting in their disassembly only when engulfed by endocytosis [73]. On the other hand, which was already mentioned, polysaccharides have the ability to interact with the mucosal layer padding the rectal lumen interior. In a few studies, Hua et al. [30] and Rathi et al. [74] reported that nanoparticles applied in liquid, solid, or semi-solid formulations for rectal administration have increased encapsulated drug half-life, bioavailability, and adsorption. Even though the number of studies in the area of DDSs for rectal administration is increasing, the evaluation of many formulations is restricted to in vitro (Caco-2 cell line) and ex vivo conditions. That vast gap in animal-based studies makes rectally administered nanoparticles far from clinical trials and should be considered as the priority to be filled.

Parenteral administration can be divided into multiple routes of drug introduction, e.g., subcutaneous (in the fatty tissue), intramuscular (into skeletal muscles), and intravenous (superficial or deep veins) [12]. Widespread among the whole human body, the closed blood circulation system is responsible for distributing oxygen and nutrients to each tissue and retaking toxic by-products. Thanks to its abundance and role, blood is a great medium to transfer pharmaceutically active substances. The smallest blood vessels’ diameter is ~5 µm [75]. Thus, DDSs for IV medication delivery should not exceed this size to distribute APIs effectively. In this sense, nanometric drug carriers exhibit a great advantage. De Jong et al. [76] performed a pharmacodynamic (PD) study on animal rat models, resulting in size-dependent NPs’ distribution among different body organs while a nanoformulation was administered intravenously. Although IV drug administration is the most common of the parenteral routes, it is also associated with a high risk of health alteration. When considering nanometric DDSs for injection, possible adverse effects should be investigated (e.g., haemolysis, thrombosis, and opsonisation) [77]. The toxicological impact of nanoparticles administered intravenously is highly connected to their surface charge and size. Although NPs possessing positive zeta potential exhibit stronger adhesion, they are considered to be more haemolytic due to the destabilisation of the red blood cell membrane [78]. The effect of nanoparticle-originated haemolysis can be easily tested in vitro, and should be an inherent part of studies on new nanometric DDSs [79]. As blood plasma is rich in multiple types of peptides and proteins, it is inevitable for NPs not to undergo opsonisation. This effect is another crucial issue that should be tested, even prior to haemolysis studies, as NPs interact with red blood cells through the protein corona rather than their surface [78]. This was confirmed by Barshtein et al. [80], as they observed the decrease in haemolytic activity due to the presence of an albumin corona created on polymeric nanoparticles. The analysis of pharmacokinetic studies for nanoparticles loaded with chemotherapeutic drugs published recently by Abdifetah and Na-Bangchang revealed that NPs improve the PK profiles due to the escape of first-pass hepatic metabolism, decrease in drug elimination by the reticuloendothelial-associated organs, and inhibition of P-gp efflux pump, among other ways [81]. An excellent review on biodistribution and excretion studies of intravenously injected nanoparticles was published in 2022 by Skotland T. et al. [82]. In the second part of the article, the authors exhaustively discussed the correlation between the size and mechanism of renal excretion. Only very small nanoparticles undergo effective clearance, limited by the 12 nm slit in the glomerular basement membrane [83]. It needs to be mentioned that the addition of targeting molecules (e.g., antibodies) to the NP surface will slightly increase the accumulation of the nanoformulation and encapsulated drug in tumour tissue [82,84]. A considerable debate was also raised around the enhanced permeability and retention (EPR) effect, the supposed mechanism for passive NP tumour targeting. However, there is still no coherent conclusion on that topic [85,86].

No matter which route of administration is selected for nanoformulation drug delivery, there is a necessary set of experiments to be carried out. First and foremost, as for any resultful drug delivery system (efficient loading capacity and loading efficiency), biocompatibility and cytotoxicity should be tested. The next fundamental aspect is to study the stability of the nanoparticles (as a solid powder, in solution, or suspension). One should take into consideration the evaluation of absorption mechanisms and analysis of DDS behaviour in the potential surroundings (e.g., interaction with mucus or secreted biomolecules, the influence of local pH, etc.).

## 3. Polish Research on Polysaccharide Nanoparticles and Their Administration Routes

**Figure 4:**
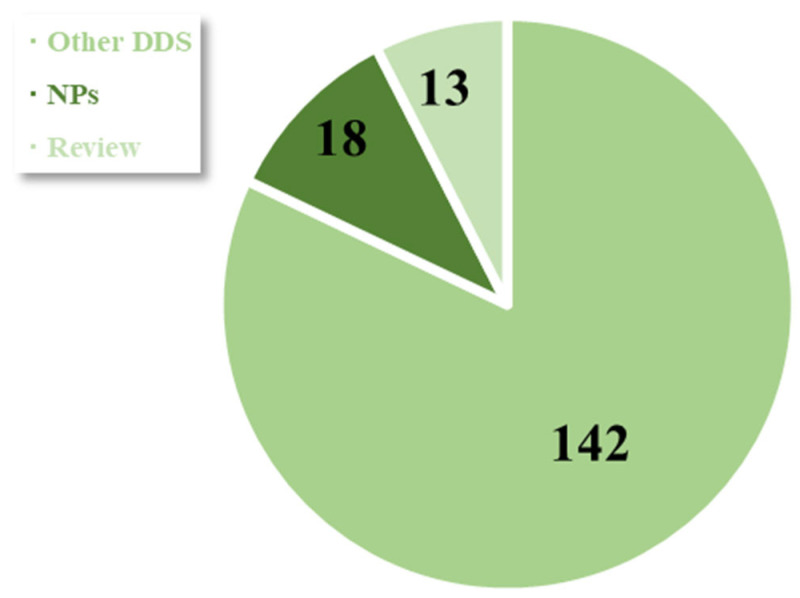
Pie chart presenting number of Polish-affiliated articles on polysaccharide drug delivery systems (NPs—research on nanoparticles; review—review articles; other DDSs—articles dedicated to films, micro- and macroparticles, gels, etc.).

We briefly screened scientific databases (PubMed, NCBI, Scopus, and Elsevier) for articles affiliated with Polish contributors published in the years 2016–2023 and containing the keywords polysaccharide, alginate, carrageenan, cellulose, dextran, gellan gum, hyaluronic acid, levan, or starch together with one of the following: nanoparticles or drug delivery. As a result, we obtained 173 publications altogether (Figure 4). Further selection was based on the first or corresponding authors with Polish affiliation and the scope of the conducted research (polysaccharide nanoparticles), so that the article number decreased to 31, including 13 review articles. Then, research papers were categorised by polysaccharide type, and are briefly described in the below sections.

As a result, we did not find any research on carrageenan, cellulose starch, or gellan gum as the primary material for NP synthesis. Only a few individual articles were dedicated to nanoparticles made of hyaluronic acid, or levan; thus, those studies were described in the last section titled ‘Nanoparticles made of other polysaccharides’. It is also essential to inform the readers that the majority of publications presented the use of polysaccharides only as an external coating for biocompatibility improvement.

### 3.1. Alginate-Based Nanoparticles

Alginic acid (AG), also called alginate or algin, is a saccharide-based polymer where β-D-mannuronate and α-L-guluronate subunits are linked via a β-1,4 glycosidic bond, forming a repetitive polymer strand. Due to carboxylic acid side groups, this seaweed-origin water-soluble polymer exhibits a strong pH-responsive tendency, making it a valuable biopolymer for DDSs. Thus, AG-based nanomaterials have tuneable permeability as well as polymer degradation properties. Moreover, the chelating ability toward divalent cations (e.g., calcium) opens a facile way for the nano delivery system preparation. The anionic character of alginate allows the electrostatic interaction with cationic molecules, both small molecules (e.g., aromatic amines) and polymers (e.g., chitosan).

The preparation of a stimuli-responsive alginate/chitosan ciprofloxacin DDS for gastrointestinal delivery was described in the work of Agnieszka Kyzioł et al. [87]. They incorporated a two-step preparation process of core–shell nanoparticles consisting of a solid crosslinked alginate core with calcium ions via an ionotropic gelation process and a hydrogel chitosan shell at pH 4.5. Core–shell nanoparticles of a size ca. 240 nm with zeta potential equal to +14 mV, with a core of ca. 160 nm with −32 mV, were obtained. The loading capacity (LC) of ciprofloxacin was reported to be 47.3 ± 4.8% after 120 h of incubation in PBS (pH = 7.4, 37 °C), based on the spectrometric method (absorbance measured at 277 nm). The concentration of the entrapped antibiotic was calculated according to the calibration curve. The proposed nanoparticles were tested in the conditions mimicking the GIT (pH: 1.2, 6.4, and 7.4), subsequently obtaining pH-dependent kinetics. At the lowest pH, the drug release mechanism is due to Fick’s diffusion, while in the case of the higher pHs it is due to anomalous non-Fick transport (pH = 6.8) and case II transport (zero-order kinetics; pH = 7.4). Moreover, the chitosan shell was found to be an effective barrier hindering drug release, preserving the drug from the acidic stomach environment. This study shows an adaptable platform for the sustainable release of antibiotics for bacterial infection defence.

Combining therapeutic activity with diagnostic capabilities is a base ground for the theragnostic field. Podgórna et al. [88] harnessed a similar approach to build a system containing a gadolinium derivative for targeted organ delivery. The nanoparticles were prepared by a reverse microemulsion method employing toluene as an oil phase and water. As a gadolinium precursor, gadolinium (III) chloride hexahydrate was used at a concentration of 3.5%. Mixing the gadolinium emulsion with the alginate emulsion caused the gelation process and nanoparticle formation. The model molecule, fluorescent rhodamine B, was used to mimic the therapeutic payload. The dye entrapment was monitored via the spectrofluorometric technique, with an excitation wavelength of 520 nm. Subsequently, several alternating layers of chitosan and alginate were grafted, forming 115 nm nanoparticles after the fifth layer with a zeta potential of ca. −30 mV—characterised by dynamic light scattering (DLS). The cell viability examined by the 3-(4,5-dimethylthiazol-2-yl)-2,5-diphenyltetrazolium bromide (MTT) test confirmed the lack of cytotoxic character for the formed nanostructures.

The AG crosslinking by divalent metal ions is one of the most used synthetic ways to obtain a nanometric system for drug delivery. Jaromin et al. [89] successfully incorporated this strategy to form the transdermal nano-DDS for the ebselen drug. The nanoparticles were prepared under sonication through the oil-in-water emulsification and gelation process. The oil phase consisted of Labrafac Lipophile WL1349, Span 80, Transcutol HP, and ebselen, while the water phase was an alginate solution (0.6 mg/mL). The formed oil-in-water emulsion achieved using the probe-type ultrasonic cell disruptor was subsequently gelated by adding 0.5 mL of CaCl_2_ solution in water (0.7 mg/mL) for 3 min. The obtained nanoparticles were purified by dialysis against water (MWCO 25 kDa). The nanoparticle size was found to be in the range of 172 ± 1 to 204 ± 1 nm with zeta potential values of −15.4 ± 0.1 to −17.1 ± 0.9 mV dictated by the presence of negatively charged anionic alginate residues. The ebselen encapsulation efficiency determined by the RP-HPLC was found to be in the range of 26.9 ± 1.2% for 172 nm nanoparticles up to 45.1 ± 2.1% for nanoparticles 204 nm in diameter. These values were directly associated with the amount of Transcutol HP solvent used. The MTT assay confirmed good biocompatibility of the formed nanosystems, with cell viability above 88%.

### 3.2. Chitosan-Based Nanoparticles

Another abundant bio-originated polysaccharide is chitosan (CHO). It is a biopolymer originating from chitin and produced in the deacetylation process. CHO comprises repeating units of ᴅ-glucosamine and N-acetyl-ᴅ-glucosamine subunits connected via β1 → 4 bonds in different configurations and frequencies. As chitin is the second (after cellulose) most abundant naturally occurring polymer biosynthesised by fungi or present in the animals’ exoskeleton, one could consider CHO an excellent biomaterial for various applications. However, some limitations are connected with the cost-effectiveness of its production process [90]. The outstanding property of CHO compared to other natural polysaccharides is its cationic character, which is responsible for its mucoadhesive properties [91].

Even though chitosan is the polysaccharide frequently mentioned in articles published by authors with Polish affiliation and with ‘drug delivery’ as a keyword (almost 70 positions), a considerable percentage is dedicated to films or microparticles rather than nanoparticles, and it is barely used as a basal material for NP synthesis. Still, it is highly preferred as a coating or stabiliser.

As mentioned previously (in the ‘Alginate-based nanoparticles’ section), Kyziol A. et al. [87] prepared alginate nanoparticles (ca. 240 nm) that gained a positive surface charge (+14 mV) due to the CHO coating on their surface. The authors noticed that drug release from the prepared nanoformulation was highly affected due to the non-specific interaction of ciprofloxacin carboxylic groups with positively charged CHO.

An interesting work published by Bazylinska U. and Saczko J. explores nucleic acid delivery systems for anticancer gene therapy [92]. CHO was used as an outer layer of synthetic quaternary surfactant-originated nanoemulsion. For that purpose, deoxyribonucleic acid (DNA) sodium salt and chitosan were adsorbed alternately on the oil–core nanoemulsion surface. The optimal ratio of polyelectrolytes (CHO and DNA) and the nanoemulsion were experimentally examined according to surface ζ potential. As a result, they obtained nanocapsules with diameters ranging from 101 nm to 119 nm, a PDI lower than 0.3, and a ζ potential of ca. +40 mV. When cytotoxic activity was tested on three malignant cancer cell lines via the MTT test (breast carcinoma (MCF-7/WT), skin melanoma (MEWO), and epithelial lung adenocarcinoma (A549)), the outcome was that NPs are promising nanocarriers for nucleic acid delivery as well as for cancer diagnostics.

Another study incorporating CHO in the structure of nanoparticles showed high encapsulation efficiency (over 90%) of the viral vector [93]. The NPs’ core was built of bacterial poly-γ-glutamic acid (γ-PGA) and CHO crosslinked with adenovirus particles. The applied synthesis was driven by an ionic interaction, and its main advantages are attributed to the lack of organic solvents and surface modification avoidance. No HEK293 cell line proliferation inhibition was measured in the presence of NPs up to 5 mg/mL, confirming its biocompatibility.

In 2019 in *Nanomaterial Journal*, Janus L. et al. [94] described the synthesis and characterisation process of chitosan-based carbon quantum dots (QDs) as a nanomaterial for diagnostics, controlled drug delivery, or cell-labelling purposes. CHO-based QDs were prepared in a microwave radiation field (300 W) in acidic conditions with simultaneous functionalisation by amino acids (lysine, glutamic acid, and cysteine) and casein hydrolysate N-doping. Fourier Transform Infrared Spectroscopy confirmed the obtained products. After fluorescence studies that demonstrated the photoluminescence properties of the obtained CHO-QD, quantum yields were determined (2.3–11.5%). Unexpectedly, the highest yield was noticed for the sample prepared from chitosan modified with lysine and carbonised for 3 min. Mean diameters were measured by DLS (5–14 nm). The final step of the research was a cytotoxic study which revealed that the obtained nanomaterial was not inhibiting the proliferation of human dermal fibroblasts (HDF), according to an XTT (2,3-Bis-(2-Methoxy-4-Nitro-5-Sulfophenyl)-2H-Tetrazolium-5-Carboxanilide) assay.

Piosik E. et al. [95] investigated the interactions of CHO-coated magnetite (Fe_3_O_4_) NPs with a cell membrane model. Although the research was filling the critical gap in the field of interactions of magnetic nanoparticles covered with a bioactive polymer with biological membranes, the synthesis and properties of nanomaterial were not the aims of the paper’s discussion. The authors declared that the size of the obtained particles was uniform (29 nm) in the formed aggregates, measured by TEM and SEM analysis.

In the study presented by Janik-Hazuka M. et al. [96], amphiphilic derivatives of chitosan and hyaluronic acid were used to prepare stable nanoemulsions for oleic acid (OA) and corn oil (CO) delivery. DLS studies revealed that the emulsion nanocapsules’ size was 352–366 nm and found a negative zeta potential of −20 mV up to over −40 mV for anionic chitosan coated vesicles. It was proved that the obtained derivatives efficiently stabilise OA and CO nanoemulsions. In vitro studies were conducted to evaluate the applicability of a nanoemulsion for anticancer therapy. It was noted that for the emulsion stabilised by a chitosan derivative, the effective cytotoxic concentration of OA was significantly higher than for emulsions stabilised by hyaluronic acid derivatives.

A recent work in the scope of this section was published by Brzezinski et al., on September 2022 [97]. Researchers investigated an injectable nanoformulation containing oleogels and tenofovir alafenamide–chitosan nanoparticles prepared by spray drying using 1% acetic acid as a solvent. Particle sizes were measured using SEM photographs and *ImageJ* software, ranging from ca. 100 nm to over 500 nm. The in vitro (PBS, pH 7.4) drug release profile was examined as best fitting to the Weibull model, and Fick’s law of diffusion was attributed as a predominantly involved mechanism. What was interesting was that the drug release decreased inversely proportional to particle size. Cytotoxicity studies (MTT assay) showed that cell viability (mouse fibroblasts, L929) was over 70% for concentrations up to 750 μg/mL. The authors summarised that the TAF–chitosan nanoparticles incorporating oleogels are a promising dosage form for prolonged drug release without significant toxic reactions.

### 3.3. Dextran-Based Nanoparticles

A biotechnologically produced biopolymer, dextran (DEX), is a family of bacterial polysaccharides built of contiguous ᴅ-glucose units linked via α-(1→6) glycosidic bonds with several branches in the α-(1→3) position. The most abundant dextran in the market is the one obtained from the *Leuconostoc mesenteroides* NRRL B-512F strain (up to 5% of the overall branching) [98]. It is FDA approved, and is a water-soluble polysaccharide with a wide range of medical applications (e.g., blood plasma expander, antithrombotic agent, and rheological modifier) [99].

Wasiak I. et al. [100] published the first Polish-affiliated work on nanoparticles incorporating dextran as a stable backbone. In the first step, polysaccharide was oxidised with sodium meta periodate (NaIO_4_), yielding a poly-aldehyde derivative of dextran (PAD). Different oxidation ratios were evaluated, and the efficiency of NAIO_4_ was estimated to produce 1.2–1.5 aldehyde groups per one sodium meta periodate molecule. PAD was subsequently substituted with amine coiling agents or doxorubicin (DOX) with Schiff base formation. The encapsulation efficiency of DOX was calculated and exceeded 92%. What was exciting was that the authors did not observe the undesired burst effect during kinetic release studies from DOX-loaded nanoparticles in different pH conditions. The size of nanoparticles was inversely correlated with the polysaccharide molecular weight, and the NPs’ composition was estimated to contain ca. 10 polymer chains. In vitro viability studies conducted on a HeLa cell line confirmed the biocompatibility of the obtained nanomaterial. Finally, the confocal micrographs revealed that DOX was released inside the living cells, which justified the assumption that the prepared nanoformulation can be further studied in vivo.

Another study by Bazylinska U. et al. [101] proposed dextran utilisation as one of the polyelectrolytes toward layer-by-layer nanocapsule fabrication. As DEX was not the basal substrate for the NP synthesis, the article was out of the scope of our review. The haemolytic activity studies that the authors described deserve an extra comment, as they were mentioned to be highly important in the fundamental research on DDSs.

In 2021, Janczewska M. et al. from NanoThea Inc., in cooperation with Ciach T. from the Warsaw University of Technology, synthesised NPs based on dextran conjugated with an inhibitor of prostate-specific membrane antigen (PSMA), Glu-Ureido-Lys, and DOTA for a radiotherapeutic application [102]. In the first approach, DEX was oxidised with metaperiodate. Subsequently, PAD was substituted with an amine group of lysine and 1,10-diaminodecane dihydrochloride and double bonds were reduced, yielding the NPs (150 nm). Another presented synthesis route toward DEX modification employed trichlorotriazine (TCT) as a conjugation agent. The radiochemical yield of the applied DOTA chelator was close to 100% when TCT was applied for conjugation. Importantly, this paper tested the impact of obtained conjugates on serum stability to investigate suitability for preclinical use. None of the synthesised nanomaterials were cytotoxic to LNCaP and PC3 cell lines, indicating their potential for use as an actively targeted radiopharmaceutical or diagnostic tool.

One more work found on dextran NPs was published in 2022 by Kulikowska-Darłak A. et al. [103]. Biomaterial synthesis started with dextran oxidation, followed by the addition of dodecylamine as a coiling agent (procedure described previously by Wasiak I. et al.) An anticancer drug, doxorubicin, was also attached to PAD by the amine group in the reaction of Schiff base formation. Afterwards, PAD-based nanoparticles were successfully encapsulated in alginate microbeads, which were subsequently covered with a chitosan multilayer. The NPs’ diameters were determined using a NanoSight LM10–HS device (112 ± 5.2 nm), and their zeta potentials were measured as slightly negative, ca. −4 mV. Two different types of microspheres incorporating PAD-NPs of varying sizes were prepared (318 μm and 441 μm, respectively). No significant diameter change was detected due to the introduction of the chitosan coating, allowing for the conclusion that the layer width is nanometric. Further studies on the stability of coated microspheres revealed that the diameter is constant over 30 days of incubation in PBS at pH 7.4 and 37 °C. There was no significant difference in the NP release profile from each type of the prepared alginate microspheres. The MTT assay was checked to evaluate the cytotoxicity of the composed material. The higher cytotoxic effect was attributed to the smaller microparticles, while the cytotoxicity of bigger microspheres was defined as ‘mild’. Summarising the studies, the authors proved that alginate microspheres containing dextran-based nanoparticles could be considered a novel drug delivery system.

### 3.4. Nanoparticles Made of Other Polysaccharides

Among other polysaccharides, hyaluronic acid (HA) was incorporated in a few recent works published by Szafraniec J. et al. to produce nanometric DDSs. The first was published in 2017 [104] and described a surfactant-free preparation technique of hydrophobically modified HA-based nanocapsules. HA is a natural polymer sourced from an extracellular matrix, glycocalyx, which guarantees its biocompatibility. HA is composed of repeating units of a disaccharide, D-glucuronic acid and N-acetyl-D-glucosamine, connected via alternating β-(1 → 4) and β-(1 → 3) glycosidic bonds [105]. In the abovementioned work, HA was modified by an amidation reaction (different hydrocarbon lengths) catalysed by an EDC/NHS coupling system. The obtained product was dissolved in a 0.1 M sodium chloride aqueous solution and ultrasonicated with oleic acid, yielding a nanoemulsion (average droplet diameter of ca. 350 nm). Interestingly, although the zeta potential was relatively constant in time (up to 14 days), the average diameter of droplets varied with different intensities depending on the hydrocarbon length. In vivo biodistribution studies revealed that IV-administered nanocapsules (NCs) accumulated in the liver, lung, and kidneys. The following research on mice proved that the nanoformulation could also be effectively applied orally. Finally, the toxicity studies showed no symptoms of toxicity of the used capsules in a concentration up to 2000 mg per kg b.w. The authors also modified nanocapsules with layer-by-layer anionic and cationic chitosan incorporation. The most stable HA derivative was the one modified by dodecyl chains (degree of substitution 4.5%). Four years later, another study published by the same research group of Jagiellonian University [106] investigated curcumin administration in hypertensive rats. A nanoemulsion was prepared as described above. The NCs loaded with curcumin revealed a lower ζ potential (−17 mV) than those enclosed over the corn oil droplets (−29 mV), but the size remained stable (ca. 400 nm). Vascular delivery of single-administered NCs was confirmed by 19FNMR spectroscopy thanks to the encapsulation of perfluorooctylbromide (PFOB), although the mechanism of retention of NCs in vascular walls remains unknown. In addition, the encapsulation efficiency of PFOB in HA-based NCs was measured to be 81%. As a result, it was proven that the prepared nanoformulation improved curcumin bioavailability, reducing the required therapeutic doses. Janik-Hazuka M et al. [107] proposed a novel HA-based nanosystem for garlic oil (GO) delivery. The concept was to overcome problems with natural compounds’ poor solubility and structural instability (e.g., susceptibility to oxidation, enzymatic degradation, redox conditions, etc.). The NC preparation method did not differ from those described in previous works already discussed. Studies were expanded to investigate the DDS interaction with serum and human body fluids. The average sizes of droplets containing GO essential substances (namely diallyl disulphide, DADS, and diallyl trisulfide, DATS) were stable over 12 weeks and equal to ca. 450 nm and 600 nm, respectively. Haemolytic activity was also investigated, resulting in a lack of a significant positive effect compared to the control. The encapsulation process can enhance DADS and DATS bioavailability, retaining their anticancer properties.

A recent study published by Lewińska A. et al. [108] was dedicated to levan nanoparticles and a surfactin-stabilised nanoemulsion for topical administration. One of the major roles of dermal cosmetics is to induce and keep an appropriate hydration level. Although HA is commonly used as a moisturising agent, it can be effectively replaced by levan. Unlike other discussed saccharides, levan is built of repeating pentose units. Fructose groups are linked by β-(2 → 6)-glycosidic bonds. The biggest drawback of this polysaccharide is the difficulty of producing meaningful quantities [109]. The levan NPs were formed spontaneously in a water solution in the discussed study. The authors of this research conducted preservative screening, prepared a model matrix, and investigated stability studies. Positive results were followed with in vivo tests on ten women volunteers. That allowed authors to conclude that levan nanoparticles improved skin hydration and elasticity.

### 3.5. Concluding Remarks

Each of discussed polysaccharides were successfully applied in nanoparticle production. As briefly summarised in Table 2, the size of the obtained NPs was in the range of 100–400 nm, with one exception (diameter <20 nm for carbon-based quantum dots). As this parameter was earnestly measured and described in papers, the zeta potential was sometimes overlooked. The observed surface charge values depend highly on the polysaccharide used, with negative values for alginate, dextran, and hyaluronic acid and positive values for chitosan. That corresponds to the presence of different functional groups in polysaccharide chains. Barely any screened NPs have an absolute value of surface charge exceeding 30 mV, which could be interpreted as an indicator of low stability. Still, this characteristic is not the only one that impacts this property, which was advanced in the above studies. As those studies were designed to deliver the selected active substances to live organisms, it was vital to evaluate cytotoxicity effects. No matter which polysaccharide type was used in NP synthesis, all demonstrated sufficient biocompatibility. The most common test scientists chose for cytotoxicity assays was the MTT test, followed by the XTT test. We can find one noticeable gap in the studies mentioned above in the context of loading capacity and encapsulation efficiency data, which are valuable parameters allowing to compare different nanoformulations in terms of drug content. Only one work of the screened studies proposed NP modification introducing active targeting (PSMA conjugate). Nevertheless, there were no studies conducted on triggered drug release, which seems to be a worldwide trend in drug nanocarrier research.

## 4. Summary

In this paper, we briefly described the relationship between the drug delivery routes and the polysaccharide nanoparticles utilised as DDSs, emphasising biopolymers’ structural characteristics. The most important properties of nanoparticles in the context of DDSs and the delivery route, such as size and zeta potential, were discussed. Polysaccharides are a promising material for NP synthesis due to their biocompatibility, relatively low production costs, high potential for chemical modifications, and biodegradable character. Their hydrophilic nature allows them to act as great solubilisers for barely soluble drugs or cosmetic ingredients. The delivery routes of different active substances demand individual properties of nanocarriers, and appropriately modified polysaccharides undoubtedly bear this potential. The obtained nanomaterial can exhibit either a cationic or anionic surface to be the most accurate for enhanced interaction with the carried API or to serve excellent adhesive properties with target tissues. DDSs also improve biodistribution due to their protective character against enzymatic degradation or harsh environmental conditions. On the other hand, a span of molecular weights of commercially available biopolymer samples, challenging chemistry, limited or lack of solubility in organic solvents, and, in some cases, slow enzymatic degradation may be significant drawbacks when considering polysaccharides as payload carriers.

The discussed studies on polysaccharide-based nanoparticles strongly differed in their complexity and scope. Even though, in most cases, the authors suggested the route of administration, there was a lack of direct comment on the potential benefits or drawbacks of the applied DDS. We read about intravenous, oral, and transdermal NP administration. Few experimental procedures were designed and proposed to investigate the effect of a possible impact of physiological conditions on nanoformulation properties and vice versa. In light of this, we feel obliged to encourage researchers to expand the scope of conducted studies toward pharmacokinetic properties and, if possible, in vivo testing. As the content of this review is restricted to research articles of authors with Polish affiliation, the readers should remember it is only a drop in the ocean of articles dedicated to polysaccharide-based nanoparticles. However, the discussed studies do not stand out from the world trends in scope and complexity. Undoubtedly, in the described papers, it should be emphasised that polysaccharide-based nanoparticles have great potential to adjust the particle size and surface charge.

## 5. Future Prospects

**Figure 5:**
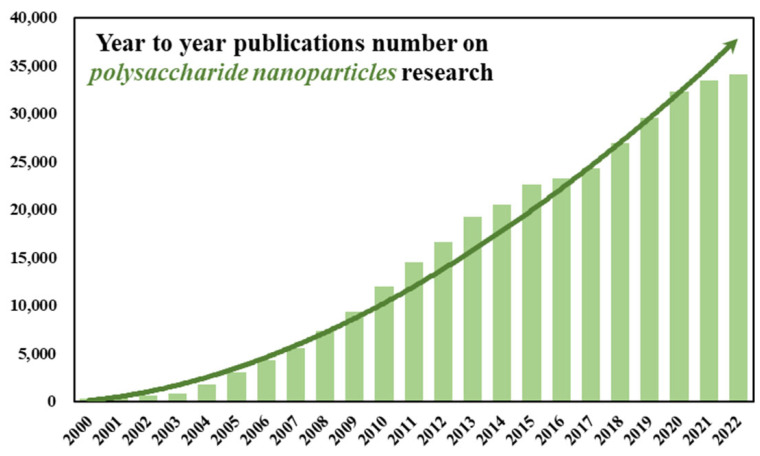
Number of articles on polysaccharide nanoparticles published from 2000 to 2022 according to PubMed and NCBI [111].

Polysaccharide nanoparticles are undoubtedly an interesting solution in the context of drug delivery systems. According to statistics in the Scopus database, scientific papers on this subject have been published in increasing number over recent years (Figure 5). Polish research centres are constantly exploring the field of DDSs, but there are few publications on nanoparticles. This gap indicates an interesting research niche that may be filled in the coming years. There are still no registered medicinal formulations on the market that use polysaccharide-based nanoparticles as drug carriers, despite their many advantages, such as biocompatibility, biodegradability, and relatively low cost of production.

The research papers discussed in the above sections are characterised by high-quality research and a multidisciplinary approach. Nevertheless, the postulated route of administration of nanoformulations is too rarely specified in the publications on polysaccharide-based nanoparticles. Scientists need to consider each delivery route’s advantages and appropriately design the nanometric DDS properties to the purpose. At this point, it is also worth noting the need to conduct cytotoxicity studies on cell lines using XTT tests [110] or apply more than one method to evaluate the results. In this context, not only the cytotoxicity of DDS should be considered, but also any other adverse effects possible to be predicted (e.g., native tissue pH destabilisation and vice versa effect of pH on NP structure and properties). Each route of administration means different types of biological, chemical, and physical barriers to overcome, which should be thoroughly explored in the planned studies. Moreover, to ensure the ease of comparison between different types of nanoparticles and various procedures, in addition to the size and surface charge, parameters such as encapsulation efficiency (EE) and loading capacity (LC) should be investigated and given in the main text of the article. In the case of conducting primary research, it is worth paying attention to the mechanisms of interaction of nanoparticles (e.g., simple adhesion or the molecular-level ligand–receptor interactions) with cells corresponding to those that will come into contact with the applied nanoparticles.

Another important issue is the study of the mechanisms of interaction of nanoparticles with cells (type of endocytosis and distribution of nanoparticles in the cell). An exciting discovery of recent years was the increased cytotoxic activity of nanoparticles made of Plutonic L61 unimers through additional mechanisms of blocking drug efflux transporters [112]. This indicates a scientific niche that needs to be taken into account in the context of future research on nanoparticles, especially those of a micellar nature, which undergo disassembly. Notably, polysaccharide-based nanoparticles’ advantages exceed the chemical modification challenge. Despite the need for special attention during synthesis, purification, and characterisation, their bio-properties open new branches of nano-medicine science. The conducted research on the cytotoxicity of polysaccharide-based nanoparticles and several in vivo studies indicated their biocompatibility, which can be seen as a huge advantage toward applicability in the pharmaceutical industry. Unfortunately, we found only two patents describing the preparation and use of polysaccharide-based nanoparticles corresponding to discussed scientific discoveries, which discourages companies from exploring this area. Undoubtedly, polysaccharide-based nanoparticles are prominent carriers with a great potential to deliver the active substance efficiently and selectively, but there is still a lot of work to be conducted.

## Figures and Tables

**Table 2:** Comparison of the most important nanoparticle characteristics (based on reviewed research articles).

Polysaccharide	Size (nm)	ζ Potential (mV)	LC (%)/EE (%)	Payload	Cytotoxicity Test	Proposed Administration Route	Ref.
ALG	160	−32	n/d	Ciprofloxacin	n/d	oral	[87]
ALG_CHO	240	+14	74.6 (LC)		n/d	oral	
Gd_ALG	115	−30	10% (EE)	Rhodamine b	MTT	n/d	[88]
ALG	172 to 204	−17 to −15	26.9 to 45.1 (EE)	Ebselen	MTT	topical	[89]
CHO	101 to 119	+43	n/d.	DNA	MTT	n/d	[92]
PGA_CHO	485	−23.7	92 (EE)	Adenovirus	MTT	n/d	[93]
CHO_QD	5 to 14	n/d	n/d	n/d	XTT	n/d	[94]
HA_CHO	366	−41.3	n/d	Oleic acid	XTT	n/d	[96]
CHO	315	n/d	n/d	TAF	MTT	n/d	[97]
DEX	100 to 140	n/d	92 (EE)	Doxorubicin	MTT	n/d	[100]
DEX_CHO	120	−44 to + 41	5.8 (LC)/96 (EE)	Daunorubicin	MTT	intravenous	[101]
DEX	80 to 150	−10 to −3	n/d	n/d	XTT	n/d	[102]
DEX	112	−3.9	n/d	Doxorubicin	MTT	n/d	[103]

Abbreviations used: ALG, alginate; CHO, chitosan; DEX, dextran; Gd, gadolinium; HA, hyaluronic acid; PGA, poly-gamma-glutamic acid; QD, quantum dots; TAF, tenofovir alafenamide; n/d, no data.

## Data Availability

Not applicable.

## References

[1] Ranade V.V., Hollinger M.A. (2004). Drug Delivery Systems.

[2] Joudeh N., Linke D. (2022). Nanoparticle classification, physicochemical properties, characterization, and applications: A comprehensive review for biologists. J. Nanobiotechnol..

[3] Liu J., Willför S., Xu C. (2015). A review of bioactive plant polysaccharides: Biological activities, functionalization, and biomedical applications. Bioact. Carbohydr. Diet. Fibre.

[4] Xue H., Wang W., Bian J., Gao Y., Hao Z., Tan J. (2022). Recent advances in medicinal and edible homologous polysaccharides: Extraction, purification, structure, modification, and biological activities. Int. J. Biol. Macromol..

[5] Horkay F., Douglas J.F., Raghavan S.R. (2021). Rheological Properties of Cartilage Glycosaminoglycans and Proteoglycans. Macromolecules.

[6] Li S., Xiong Q., Lai X., Li X., Wan M., Zhang J., Yan Y., Cao M., Lu L., Guan J. (2016). Molecular Modification of Polysaccharides and Resulting Bioactivities. Compr. Rev. Food Sci. Food Saf..

[7] Khan Y., Sadia H., Ali Shah S.Z., Khan M.N., Shah A.A., Ullah N., Ullah M.F., Bibi H., Bafakeeh O.T., Khedher N.B. (2022). Classification, Synthetic, and Characterization Approaches to Nanoparticles, and Their Applications in Various Fields of Nanotechnology: A Review. Catalysts.

[8] Patra J.K., Das G., Fraceto L.F., Campos E.V.R., Rodriguez-Torres M.D.P., Acosta-Torres L.S., Diaz-Torres L.A., Grillo R., Swamy M.K., Sharma S. (2018). Nano based drug delivery systems: Recent developments and future prospects. J. Nanobiotechnol..

[9] Karavelidis V., Karavas E., Giliopoulos D., Papadimitriou S., Bikiaris D. (2011). Evaluating the effects of crystallinity in new biocompatible polyester nanocarriers on drug release behavior. Int. J. Nanomed..

[10] Plucinski A., Lyu Z., Schmidt B.V.K.J. (2021). Polysaccharide nanoparticles: From fabrication to applications. J. Mater. Chem. B.

[11] Azman M., Sabri A.H., Anjani Q.K., Mustaffa M.F., Hamid K.A. (2022). Intestinal Absorption Study: Challenges and Absorption Enhancement Strategies in Improving Oral Drug Delivery. Pharmaceuticals.

[12] Jain K.K. (2020). An Overview of Drug Delivery Systems.

[13] Jedrzejas M.J. (2000). Structural and functional comparison of polysaccharide-degrading enzymes. Crit. Rev. Biochem. Mol. Biol..

[14] Keemink J., Bergström C.A.S. (2018). Caco-2 Cell Conditions Enabling Studies of Drug Absorption from Digestible Lipid-Based Formulations. Pharm. Res..

[15] Artursson P., Karlsson J. (1991). Correlation between oral drug absorption in humans and apparent drug permeability coefficients in human intestinal epithelial (Caco-2) cells. Biochem. Biophys. Res. Commun..

[16] Florence A.T., Jani P.U. (1994). Novel Oral Drug Formulations: Their Potential in Modulating Adverse Effects. Drug Saf..

[17] Purohit T.J., Hanning S.M., Wu Z. (2018). Advances in rectal drug delivery systems. Pharm. Dev. Technol..

[18] Homayun B., Lin X., Choi H.J. (2019). Challenges and recent progress in oral drug delivery systems for biopharmaceuticals. Pharmaceutics.

[19] Hua S. (2020). Advances in Oral Drug Delivery for Regional Targeting in the Gastrointestinal Tract-Influence of Physiological, Pathophysiological and Pharmaceutical Factors. Front. Pharmacol..

[20] Li D.F., Yang M.F., Xu H.M., Zhu M.Z., Zhang Y., Tian C.M., Nie Y.Q., Wang J.Y., Liang Y.J., Yao J. (2022). Nanoparticles for oral delivery: Targeted therapy for inflammatory bowel disease. J. Mater. Chem. B.

[21] Zha S., Wong K.L., All A.H. (2022). Intranasal Delivery of Functionalized Polymeric Nanomaterials to the Brain. Adv. Healthc. Mater..

[22] Gote V., Sikder S., Sicotte J., Pal D. (2019). Ocular drug delivery: Present innovations and future challenges. J. Pharmacol. Exp. Ther..

[23] Jansook P., Hnin H.M., Loftsson T., Stefánsson E. (2021). Cyclodextrin-based formulation of carbonic anhydrase inhibitors for ocular delivery—A review. Int. J. Pharm..

[24] Subrizi A., del Amo E.M., Korzhikov-Vlakh V., Tennikova T., Ruponen M., Urtti A. (2019). Design principles of ocular drug delivery systems: Importance of drug payload, release rate, and material properties. Drug Discov. Today.

[25] Prausnitz M.R., Langer R. (2008). Transdermal drug delivery. Nat. Biotechnol..

[26] Dimitriu P.A., Iker B., Malik K., Leung H., Mohn W.W., Hillebrand G.G. (2019). New insights into the intrinsic and extrinsic factors that shape the human skin microbiome. MBio.

[27] Zhang Y.B., Xu D., Bai L., Zhou Y.M., Zhang H., Cui Y.L. (2022). A Review of Non-Invasive Drug Delivery through Respiratory Routes. Pharmaceutics.

[28] Forest V., Pourchez J. (2022). Nano-delivery to the lung-by inhalation or other routes and why nano when micro is largely sufficient?. Adv. Drug Deliv. Rev..

[29] Javadzadeh Y., Yaqoubi S. (2017). Therapeutic Nanostructures for Pulmonary Drug Delivery.

[30] Hua S. (2019). Physiological and pharmaceutical considerations for rectal drug formulations. Front. Pharmacol..

[31] Gundloori R.V.N., Singam A., Killi N. (2018). Nanobased Intravenous and Transdermal Drug Delivery Systems.

[32] Shim S., Yoo H.S. (2020). The Application of Mucoadhesive Chitosan Nanoparticles in Nasal Drug Delivery. Mar. Drugs.

[33] Cunha S., Amaral M.H., Sousa Lobo J.M., Silva A.C. (2017). Lipid nanoparticles for nasal/intranasal drug delivery. Crit. Rev. Ther. Drug Carr. Syst..

[34] Kim D.-D., Ehrhardt C., Kim K.-J. (2008). Drug Absorption Studies. Drug Absorption Studies.

[35] Verma P., Patel V., Prashar N., Kumar V., Chaudhary H. (2016). Nasal (In-Situ) Gel (Phenylepherine HCl) for Allergic Rhinitis Congestion Treatment: Development and Characterization. Am. J. PharmTech Res..

[36] Zhong H., Chan G., Hu Y., Hu H., Ouyang D. (2018). A comprehensive map of FDA-approved pharmaceutical products. Pharmaceutics.

[37] Berner B., John V.A. (1994). Pharmacokinetic Characterisation of Transdermal Delivery Systems. Clin. Pharmacokinet..

[38] Alkilani A.Z., McCrudden M.T.C., Donnelly R.F. (2015). Transdermal drug delivery: Innovative pharmaceutical developments based on disruption of the barrier properties of the stratum corneum. Pharmaceutics.

[39] Mitragotri S., Anissimov Y.G., Bunge A.L., Frasch H.F., Guy R.H., Hadgraft J., Kasting G.B., Lane M.E., Roberts M.S. (2011). Mathematical models of skin permeability: An overview. Int. J. Pharm..

[40] Zhang Q., Grice J., Wang G., Roberts M. (2009). Cutaneous Metabolism in Transdermal Drug Delivery. Curr. Drug Metab..

[41] Jeong W.Y., Kwon M., Choi H.E., Kim K.S. (2021). Recent advances in transdermal drug delivery systems: A review. Biomater. Res..

[42] Jiménez-Rodríguez A., Guardado-Félix D., Antunes-Ricardo M. (2022). Challenges and Strategies for Topical and Transdermal Delivery of Bioactive Peptides. Crit. Rev. Ther. Drug Carr. Syst..

[43] Som I., Bhatia K., Yasir M. (2012). Status of surfactants as penetration enhancers in transdermal drug delivery. J. Pharm. Bioallied Sci..

[44] Contri R.V., Fiel L.A., Alnasif N., Pohlmann A.R., Guterres S.S., Schäfer-Korting M. (2016). Skin penetration and dermal tolerability of acrylic nanocapsules: Influence of the surface charge and a chitosan gel used as vehicle. Int. J. Pharm..

[45] Ghosh P., Milewski M., Paudel K. (2015). In vitro/in vivo correlations in transdermal product development. Ther. Deliv..

[46] Abd E., Yousef S.A., Pastore M.N., Telaprolu K., Mohammed Y.H., Namjoshi S., Grice J.E., Roberts M.S. (2016). Skin models for the testing of transdermal drugs. Clin. Pharmacol. Adv. Appl..

[47] Ibrahim M., Garcia-Contreras L. (2013). Mechanisms of absorption and elimination of drugs administered by inhalation. Ther. Deliv..

[48] Ghanem R., Laurent V., Roquefort P., Haute T., Ramel S., Le Gall T., Aubry T., Montier T. (2021). Optimizations of in vitro mucus and cell culture models to better predict in vivo gene transfer in pathological lung respiratory airways: Cystic fibrosis as an example. Pharmaceutics.

[49] Das S.C., Stewart P.J. (2016). The influence of lung surfactant liquid crystalline nanostructures on respiratory drug delivery. Int. J. Pharm..

[50] Patton J.S., Byron P.R. (2007). Inhaling medicines: Delivering drugs to the body through the lungs. Nat. Rev. Drug Discov..

[51] Ehrmann S., Schmid O., Darquenne C., Rothen-Rutishauser B., Sznitman J., Yang L., Barosova H., Vecellio L., Mitchell J., Heuze-Vourc’h N. (2020). Innovative preclinical models for pulmonary drug delivery research. Expert Opin. Drug Deliv..

[52] Mobley C., Hochhaus G. (2001). Methods used to assess pulmonary deposition and absorption of drugs. Drug Discov. Today.

[53] Rogueda P.G.A., Traini D. (2007). The nanoscale in pulmonary delivery. Part 2: Formulation platforms. Expert Opin. Drug Deliv..

[54] Labiris N.R., Dolovich M.B. (2003). Pulmonary drug delivery. Part I: Physiological factors affecting therapeutic effectiveness of aerosolized medications. Br. J. Clin. Pharmacol..

[55] Shirley M. (2019). Amikacin Liposome Inhalation Suspension: A Review in Mycobacterium avium Complex Lung Disease. Drugs.

[56] Cryan S.A., Sivadas N., Garcia-Contreras L. (2007). In vivo animal models for drug delivery across the lung mucosal barrier. Adv. Drug Deliv. Rev..

[57] Hornof M., Toropainen E., Urtti A. (2005). Cell culture models of the ocular barriers. Eur. J. Pharm. Biopharm..

[58] Varela-Fernández R., Díaz-Tomé V., Luaces-Rodríguez A., Conde-Penedo A., García-Otero X., Luzardo-álvarez A., Fernández-Ferreiro A., Otero-Espinar F.J. (2020). Drug delivery to the posterior segment of the eye: Biopharmaceutic and pharmacokinetic considerations. Pharmaceutics.

[59] Agrahari V., Mandal A., Agrahari V., Trinh H.M., Joseph M., Ray A., Hadji H., Mitra R., Pal D., Mitra A.K. (2016). A comprehensive insight on ocular pharmacokinetics. Drug Deliv. Transl. Res..

[60] Vellonen K.S., Soini E.M., Del Amo E.M., Urtti A. (2016). Prediction of ocular drug distribution from systemic blood circulation. Mol. Pharm..

[61] Tojo K. (2004). A pharmacokinetic model for ocular drug delivery. Chem. Pharm. Bull..

[62] Mandal A., Gote V., Pal D., Ogundele A., Mitra A.K. (2019). Ocular Pharmacokinetics of a Topical Ophthalmic Nanomicellar Solution of Cyclosporine (Cequa^®^) for Dry Eye Disease. Pharm. Res..

[63] Akhter S., Anwar M., Siddiqui M.A., Ahmad I., Ahmad J., Ahmad M.Z., Bhatnagar A., Ahmad F.J. (2016). Improving the topical ocular pharmacokinetics of an immunosuppressant agent with mucoadhesive nanoemulsions: Formulation development, in-vitro and in-vivo studies. Colloids Surf. B Biointerfaces.

[64] Shafaie S., Hutter V., Cook M.T., Brown M.B., Chau D.Y.S. (2016). In Vitro Cell Models for Ophthalmic Drug Development Applications. Biores. Open Access.

[65] Vasconcelos T., da Silva S.B., Ferreira D., Pintado M., Marques S. (2016). Cell-based in vitro models for ocular permeability studies. Concepts and Models for Drug Permeability Studies: Cell and Tissue Based In Vitro Culture Models.

[66] Weng Y., Liu J., Jin S., Guo W., Liang X., Hu Z. (2017). Nanotechnology-based strategies for treatment of ocular disease. Acta Pharm. Sin. B.

[67] van Hoogdalem E.J., de Boer A.G., Breimer D.D. (1991). Pharmacokinetics of Rectal Drug Administration, Part I. Clin. Pharmacokinet..

[68] Nigro N.D. (1985). Animal model for colorectal cancer. Prog. Clin. Biol. Res..

[69] Baydi Z., Limami Y., Khalki L., Zaid N., Naya A., Mtairag E.M., Oudghiri M., Zaid Y. (2021). An Update of Research Animal Models of Inflammatory Bowel Disease. Sci. World J..

[70] Nunes R., Sarmento B., Das Neves J. (2014). Formulation and delivery of anti-HIV rectal microbicides: Advances and challenges. J. Control. Release.

[71] Tsuruya A., Kuwahara A., Saito Y., Yamaguchi H., Tenma N., Inai M., Takahashi S., Tsutsumi E., Suwa Y., Totsuka Y. (2016). Major Anaerobic Bacteria Responsible for the Production of Carcinogenic Acetaldehyde from Ethanol in the Colon and Rectum. Alcohol Alcohol..

[72] Shen T.C.D., Daniel S.G., Patel S., Kaplan E., Phung L., Lemelle-Thomas K., Chau L., Herman L., Trisolini C., Stonelake A. (2021). The Mucosally-Adherent Rectal Microbiota Contains Features Unique to Alcohol-Related Cirrhosis. Gut Microbes.

[73] Manzanares D., Ceña V. (2020). Endocytosis: The nanoparticle and submicron nanocompounds gateway into the cell. Pharmaceutics.

[74] Rathi R., Sanshita, Kumar A., Vishvakarma V., Huanbutta K., Singh I., Sangnim T. (2022). Advancements in Rectal Drug Delivery Systems: Clinical Trials, and Patents Perspective. Pharmaceutics.

[75] Chenthamara D., Subramaniam S., Ramakrishnan S.G., Krishnaswamy S., Essa M.M., Lin F.H., Qoronfleh M.W. (2019). Therapeutic efficacy of nanoparticles and routes of administration. Biomater. Res..

[76] De Jong W.H., Hagens W.I., Krystek P., Burger M.C., Sips A.J.A.M., Geertsma R.E. (2008). Particle size-dependent organ distribution of gold nanoparticles after intravenous administration. Biomaterials.

[77] Sharma M. (2018). Transdermal and Intravenous Nano Drug Delivery Systems: Present and Future.

[78] Yedgar S., Barshtein G., Gural A. (2022). Hemolytic Activity of Nanoparticles as a Marker of Their Hemocompatibility. Micromachines.

[79] Neun B.W., Dobrovolskaia M.A. (2011). Method for Analysis of Nanoparticle Hemolytic Properties In Vitro. Methods Mol. Biol..

[80] Barshtein G., Arbell D., Yedgar S. (2011). Hemolytic effect of polymeric nanoparticles: Role of albumin. IEEE Trans. Nanobiosci..

[81] Abdifetah O., Na-Bangchang K. (2019). Pharmacokinetic studies of nanoparticles as a delivery system for conventional drugs and herb-derived compounds for cancer therapy: A systematic review. Int. J. Nanomed..

[82] Skotland T., Iversen T.G., Llorente A., Sandvig K. (2022). Biodistribution, pharmacokinetics and excretion studies of intravenously injected nanoparticles and extracellular vesicles: Possibilities and challenges. Adv. Drug Deliv. Rev..

[83] Adhipandito C.F., Cheung S.H., Lin Y.H., Wu S.H. (2021). Atypical renal clearance of nanoparticles larger than the kidney filtration threshold. Int. J. Mol. Sci..

[84] Goldenberg D.M. (2003). Advancing role of radiolabeled antibodies in the therapy of cancer. Cancer Immunol. Immunother..

[85] Danhier F. (2016). To exploit the tumor microenvironment: Since the EPR effect fails in the clinic, what is the future of nanomedicine?. J. Control. Release.

[86] Wilhelm S., Tavares A.J., Dai Q., Ohta S., Audet J., Dvorak H.F., Chan W.C.W. (2016). Analysis of nanoparticle delivery to tumours. Nat. Rev. Mater..

[87] Kyzioł A., Mazgała A., Michna J., Regiel-Futyra A., Sebastian V. (2017). Preparation and characterization of alginate/chitosan formulations for ciprofloxacin-controlled delivery. J. Biomater. Appl..

[88] Podgórna K., Szczepanowicz K., Piotrowski M., Gajdošová M., Štěpánek F., Warszyński P. (2017). Gadolinium alginate nanogels for theranostic applications. Colloids Surf. B Biointerfaces.

[89] Jaromin A., Zarnowski R., Piȩtka-Ottlik M., Andes D.R., Gubernator J. (2018). Topical delivery of ebselen encapsulated in biopolymeric nanocapsules: Drug repurposing enhanced antifungal activity. Nanomedicine.

[90] Dumitriu S. (2004). Polysaccharides: Structural Diversity and Functional Versatility.

[91] Narain R. (2011). Engineered Carbohydrate-Based Materials for Biomedical Applications—Polymers, Surfaces, Dendrimers, Nanoparticles, and Hydrogels.

[92] Bazylińska U., Saczko J. (2016). Nanoemulsion-templated polylelectrolyte multifunctional nanocapsules for DNA entrapment and bioimaging. Colloids Surf. B Biointerfaces.

[93] Khalil I.R., Khechara M.P., Kurusamy S., Armesilla A.L., Gupta A., Mendrek B., Khalaf T., Scandola M., Focarete M.L., Kowalczuk M. (2018). Poly-Gamma-Glutamic Acid (-PGA)-based encapsulation of adenovirus to evade neutralizing antibodies. Molecules.

[94] Janus Ł., Piatkowski M., Radwan-Pragłowska J., Bogdał D., Matysek D. (2019). Chitosan-based carbon quantum dots for biomedical applications: Synthesis and characterization. Nanomaterials.

[95] Piosik E., Klimczak P., Ziegler-Borowska M., Chełminiak-Dudkiewicz D., Martyński T. (2020). A detailed investigation on interactions between magnetite nanoparticles functionalized with aminated chitosan and a cell model membrane. Mater. Sci. Eng. C.

[96] Janik-Hazuka M., Szafraniec-Szczęsny J., Kamiński K., Odrobińska J., Zapotoczny S. (2020). Uptake and in vitro anticancer activity of oleic acid delivered in nanocapsules stabilized by amphiphilic derivatives of hyaluronic acid and chitosan. Int. J. Biol. Macromol..

[97] Narayanan V.H.B., Lewandowski A., Durai R., Gonciarz W., Wawrzyniak P., Brzezinski M. (2022). Spray-dried tenofovir alafenamide-chitosan nanoparticles loaded oleogels as a long-acting injectable depot system of anti-HIV drug. Int. J. Biol. Macromol..

[98] Konur O. (2016). Glycoscience: The Current State of the Research.

[99] Maia J., Evangelista M.B., Gil H., Ferreira L., Gil M.H. (2014). Dextran-based materials for biomedical applications. Carbohydrates Applications in Medicine.

[100] Wasiak I., Kulikowska A., Janczewska M., Michalak M., Cymerman I.A., Nagalski A., Kallinger P., Szymanski W.W., Ciach T. (2016). Dextran nanoparticle synthesis and properties. PLoS ONE.

[101] Bazylińska U., Pietkiewicz J., Rossowska J., Chodaczek G., Gamian A., Wilk K.A. (2017). Polyelectrolyte Oil-Core Nanocarriers for Localized and Sustained Delivery of Daunorubicin to Colon Carcinoma MC38 Cells: The Case of Polysaccharide Multilayer Film in Relation to PEG-ylated Shell. Macromol. Biosci..

[102] Janczewska M., Szkop M., Pikus G., Kopyra K., Świątkowska A., Brygoła K., Karczmarczyk U., Walczak J., Żuk M.T., Duszak J. (2021). PSMA targeted conjugates based on dextran. Appl. Radiat. Isot..

[103] Kulikowska-Darłak A., Stefanek A., Wasiak-Wojasińska I., Wiechecka-Ożdżyńska P., Ciach T. (2022). Hydrogel microspheres containing dextran-based nanoparticles as novel anticancer drug delivery system. Chem. Process Eng.-Inz. Chem. Proces..

[104] Szafraniec J., Błazejczyk A., Kus E., Janik M., Zajac G., Wietrzyk J., Chlopicki S., Zapotoczny S. (2017). Robust oil-core nanocapsules with hyaluronate-based shells as promising nanovehicles for lipophilic compounds. Nanoscale.

[105] Fraser J.R.E., Laurent T.C., Laurent U.B.G. (1997). Hyaluronan: Its nature, distribution, functions and turnover. J. Intern. Med..

[106] Czyzynska-Cichon I., Janik-Hazuka M., Szafraniec-Szczęsny J., Jasinski K., Węglarz W.P., Zapotoczny S., Chlopicki S. (2021). Low dose curcumin administered in hyaluronic acid-based nanocapsules induces hypotensive effect in hypertensive rats. Int. J. Nanomed..

[107] Janik-hazuka M., Kamiński K., Kaczor-kamińska M., Szafraniec-szczęsny J., Kmak A., Kassassir H., Watała C., Wróbel M., Zapotoczny S. (2021). Hyaluronic acid-based nanocapsules as efficient delivery systems of garlic oil active components with anticancer activity. Nanomaterials.

[108] Lewińska A., Domżał-Kędzia M., Kierul K., Bochynek M., Pannert D., Nowaczyk P., Łukaszewicz M. (2021). Targeted hybrid nanocarriers as a system enhancing the skin structure. Molecules.

[109] Öner E.T., Hernández L., Combie J. (2016). Review of Levan polysaccharide: From a century of past experiences to future prospects. Biotechnol. Adv..

[110] Podgórski R., Wojasiński M., Ciach T. (2022). Nanofibrous materials affect the reaction of cytotoxicity assays. Sci. Rep..

[111] PubMed National Center for Biotechnology Information. https://pubmed.ncbi.nlm.nih.gov.

[112] Hong W., Shi H., Qiao M., Zhang Z., Yang W., Dong L., Xie F., Zhao C., Kang L. (2017). PH-sensitive micelles for the intracellular co-delivery of curcumin and Pluronic L61 unimers for synergistic reversal effect of multidrug resistance. Sci. Rep..

